# RFamide-related Peptide-3 and the Trade-off between Reproductive and Ingestive Behavior

**DOI:** 10.1093/icb/icx097

**Published:** 2017-07-27

**Authors:** Jill E Schneider, Noah A Benton, Kim A Russo, Candice M Klingerman, Wilbur P Williams, Jessica Simberlund, Amir Abdulhay, Jeremy M Brozek, Lance J Kriegsfeld

**Affiliations:** 1 Department of Biological Sciences, Lehigh University, Bethlehem, PA 18015, USA; 2 Department of Psychology and The Helen Wills Neuroscience Institute, University of California, Berkeley, CA 94720, USA; 3 Department of Biological and Allied Health Sciences, Bloomsburg University, Bloomsburg, PA 17815, USA

## Abstract

Ingestive and sex behaviors are important for individual survival and reproductive success, but when environmental energy availability is limited, individuals of many different species make a trade-off, forfeiting sex for ingestive behavior. For example, food-deprived female Syrian hamsters (*Mesocricetus auratus*) forego vaginal scent marking and lordosis (sex behaviors) in favor of foraging, hoarding, and eating food (ingestive behavior). Reproductive processes tend to be energetically costly, and individual survival requires homeostasis in metabolic energy. Thus, during energetic challenges, the chances of survival are enhanced by decreasing the energy expended on reproductive processes. The entire hypothalamic-pituitary-gonadal (HPG) system is inhibited by severe energetic challenges, but comparatively little is known about the effects of mild energetic challenges. We hypothesized that (1) a trade-off is made between sex and ingestive behavior even when the level of food restriction is insufficient to inhibit the HPG system; (2) mild energetic challenges force a trade-off between appetitive ingestive and sex behaviors, but not consummatory versions of the same behaviors; and (3) the trade-off is orchestrated by ovarian steroid modulation of RFamide-related peptide 3 (RFRP-3). In other species, RFRP-3, an ortholog of avian gonadotropin-inhibitory hormone, is implicated in control of behavior in response to energetic challenges and stressful stimuli. In support of our three hypotheses, there is a “dose-response” effect of food restriction and re-feeding on the activation of RFRP-3-immunoreactive cells in the dorsomedial hypothalamus and on appetitive behaviors (food hoarding and sexual motivation), but not on consummatory behaviors (food intake and lordosis), with no significant effect on circulating levels of estradiol or progesterone. The effect of food restriction on the activation of RFRP-3 cells is modulated at the time of estrus in gonadally-intact females and in ovariectomized females treated with progesterone alone or with estradiol plus progesterone. Intracerebral treatment with RFRP-3 results in significant decreases in sexual motivation and results in significant but small increases in food hoarding in hamsters fed *ad libitum*. These and other results are consistent with the idea that ovarian steroids and RFRP-3 are part of a system that orchestrates trade-offs in appetitive behaviors in environments where energy availability fluctuates.

## Introduction

Individual survival requires cellular homeostasis in the availability of oxidizable metabolic fuels. Most organisms maintain energy homeostasis by adjusting the components of energy balance: energy expenditure (including the energy expended on reproduction), food intake, and/or the breakdown or accumulation of stored fuels. The literature is fraught with statements suggesting that food intake and body weight are “regulated” or maintained at a “set point”; however, the obligatory homeostatic variable is the availability of oxidizable fuels. Food intake and body weight are two variables that must fluctuate, sometimes drastically, to regulate the availability of fuels (Friedman 2008). Homeostasis in fuel availability is critical in the brain because elevated circulating glucose levels are rapidly neurotoxic, and insufficient glucose levels are incompatible with the neuronal function necessary for life. For similar reasons, homeostasis in the availability of oxidizable fuels must be maintained in the periphery. Elevated levels of food intake, in contrast, are often well tolerated, and the mechanisms that increase food intake are reliably stimulated by energy deficits. Less reliable are decreases in food intake that are sufficient to prevent body fat accumulation. Increased levels of body fat content can be maintained for days or months without immediate effects on mortality. In fact, body fat accumulation provides a buffer against fluctuation in environmental energy sources, and sources of energy fluctuate seasonally or unpredictably in most natural environments (Bronson 1989). Thus, overeating, food hoarding, and body fat storage provide means to anticipate future energy shortages without damage to tissues that would result from storing excess glucose in circulation. Furthermore, excess body fat accumulation has clear adaptive significance because it is critical for the energetically costly processes related to reproduction. Food intake is rarely maintained at a set level. Rather, food intake is almost always increased when energy expenditure increases, e.g., when animals are exposed to cold ambient temperatures or when mothers are feeding hungry offspring during lactation. Just as fluctuations in food intake are a necessary reality, body weight often fluctuates markedly. If animals are to survive during famine or during a drop in ambient temperature that is not accompanied by increased food availability, body weight cannot remain in homeostasis. Body weight loss due to lipolysis is a ubiquitous adaptation necessary for survival during food shortages and cold ambient temperature (Wang et al. 2006). Body weight gain over the lifespan is a common feature in humans and pets, and in laboratory and feral animals (Martin et al. 2010; Klimentidis et al. 2011). In addition to alterations in food intake and adiposity, animals alter their energy expenditure to maintain homeostasis in the availability of metabolic fuels. In summary, energy intake, storage, and expenditure are altered to (1) maintain homeostasis in the availability of oxidizable metabolic fuels, a necessity for cellular life, and (2) anticipate the energetically costly processes of reproduction and fluctuations in environmental energy availability (reviewed by Schneider et al. (2012)).

It follows that ingestive behaviors are increased or decreased by mechanisms linked to the trade-off between survival and reproductive success. In most habitats, energy availability fluctuates and reproductive success is maximized by synchronizing reproductive activity with periods of energy abundance (reviewed by Bronson (1989)). Furthermore, reproductive processes are energetically costly, particularly in female mammals that invest heavily in milk production (reviewed by Gittleman and Thompson (1988)). Thus, when environmental energy availability is limited, females of many species make trade-offs. Somewhat paradoxically, females can sometimes increase their reproductive success by inhibition of reproductive processes. For example, long-term reproductive success can be optimized if the inhibition of reproduction improves the chances of survival in energetically challenging conditions and if reproductive processes resume when conditions improve. Most research on metabolic control of reproduction has focused on obligate inhibition of the hypothalamic-pituitary-gonadal (HPG) system. For example, in a wide array of species representing most vertebrate taxa, the HPG system is inhibited by prolonged, severe food deprivation or treatment with drugs that block the utilization of metabolic fuels such as glucose and free fatty acids (reviewed by Bronson (1989); Fernandez-Fernandez et al. (2006); Hill et al. (2008); I'Anson et al. (1991); Schneider (2004); Schneider et al. (2013); Tena-Sempere (2017); Wade and Jones (2004); Wade and Schneider (1992)). Conversely, animals often forego ingestive behaviors (foraging, eating, and food hoarding) when they engage in mate searching, courtship, and copulation (reviewed by Mrosovsky (1990); Mrosovsky and Sherry (1980)). Some of these cycles of fasting and feeding occur annually, and in aquatic mammals and birds, the amplitudes of these annual fluctuations are extreme. Bull elephant seals (*Miriounga angustirostris*), fur seals (*Callorhinus ursinus*), and male king and Emperor penguins (*Aptenodytes patagonicus* and *Aptenodytes forsteri*) spend several months at sea doing little else but fishing, eating, and accumulating massive stores of adipose tissue. In the subsequent season, they fast for several weeks while they compete for territories and mate with females. Female seals continue to fast during lactation; male and female penguins continue to fast for even longer periods of time while they incubate their eggs in subzero ambient temperatures (Dewasmes et al. 1980; Baker et al. 1994; Le Boeuf and Laws 1994; Robin et al. 1998). These are extreme cycles of fasting and re-feeding, but such cycles are common in other species, although not all are so extreme in duration and amplitude. Red deer stags (*Cervus elaphus*) reduce food intake and body fat during the rut (Mitchell et al. 1976); jungle fowl (*Gallus gallus*) mothers eat little during their 20-day incubation period (Sherry et al. 1980); and garter snakes emerge from hibernation in spring and continue to fast for a 2-week breeding period followed by a period of dispersal and foraging (Cease et al. 2007; Lutterschmidt 2017). In at least some fish species, the trade-off between foraging and courtship is apparent when energy and/or territories are limiting (Abrahams 1993; Griffiths 1996; Wong et al. 2007). Decisions about whether to eat food or search for mates are determined by prior metabolic status in male roundworms, *Caenorhabditis elegans*, with a nervous system composed of only a few hundred neurons (Lipton et al. 2004; Emmons 2017). In species representing many different taxa throughout the animal kingdom, the mechanisms that control ingestive behavior are linked to the mechanisms that control reproduction (reviewed by Schneider et al. (2013)). Many different chemical messengers alter food intake when applied directly to the brain, yet none have been demonstrated to maintain so-called homeostasis in body weight and food intake. An alternative hypothesis is that the function of at least some of these chemical messengers is to optimize reproductive success by orchestrating trade-offs in reproductive and ingestive behavior in environments in which energy is constrained.

## Mild energetic challenges affect appetitive but not consummatory sex and ingestive behaviors without affecting plasma estradiol concentrations or inducing anestrus

Inspired by this perspective, the Schneider laboratory began to study the trade-off between ingestive and sex behavior over the estrous cycle in female Syrian hamsters (*Mesocricetus auratus*). In their native habitat on the border of Syria and Turkey, this species avoids predation by living solitarily in underground burrows, and by emerging to forage for about 90-min per day only at dawn and dusk (Gattermann et al. 2008). We therefore speculated that, during this short time period, Syrian hamsters are likely to make trade-offs in ingestive and sex behavior. Wild hamsters have been observed traveling long distances (one hundred or more yards) to their food sources where they gather large quantities of food in their impressive cheek pouches before carrying it to the safety of their burrow (Johnston, personal communication). Furthermore, hamsters have an interesting coordinated adaptation to the aftermath of food deprivation. Rather than increasing food intake, they increase food hoarding (Phillips et al. 1989; Buckley and Schneider 2003) and survive by decreasing overall energy expenditure and by mobilizing lipids from adipose tissue (Silverman and Zucker 1976; Borer et al. 1985). This suggests that vigilant food hoarding is required to anticipate future energy demands, and opting to forego food hoarding to engage in sexual exploits would entail considerable risk. Thus, in our laboratory, we examined both food hoarding and sex behavior at the onset of the dark phase of the photoperiod in females that live in and emerge from a simulated burrow (Schneider et al. 2007; Klingerman et al. 2010).

### Syrian hamster appetitive behaviors

**Fig. 1:**
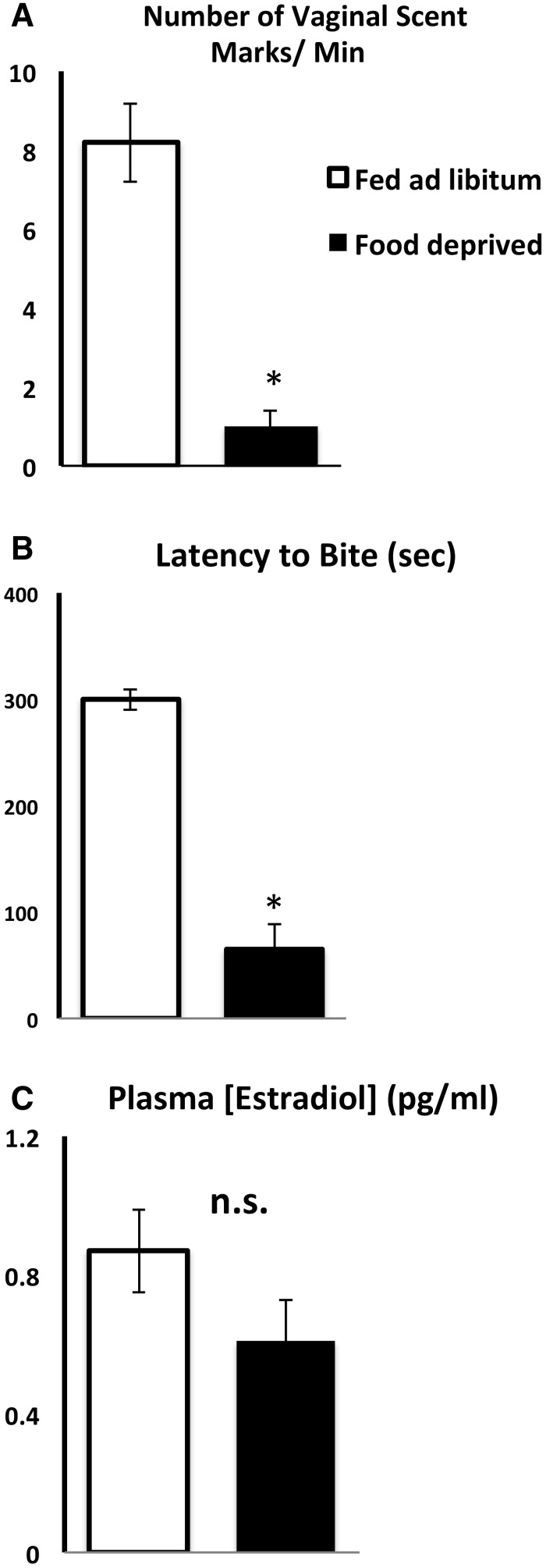
Mean and standard error of the mean for (**A**) the number of vaginal scent marks that females made in 15 min when exposed to a sexually-experienced male hamster (**B**) the latency to inflict aggressive bites on a sexually-experienced male hamster, and (**C**) the plasma estradiol concentrations. All variables were measured just after lights out on the third day of the estrous cycle. *Significantly different from *ad libitum* at *P* < 0.05 (Adapted from Schneider et al. 2007).

At the time of estrus, female hamster sex behavior in response to a male is quite reflexive and “all-or-none.” Therefore, it might be assumed that female hamsters have little flexibility in their decisions about mating. The peri-ovulatory levels of estradiol and progesterone and stimuli from an adult, sexually-experienced male typically guarantee that the consummatory sex behavior, lordosis, will occur unless the female has been subjected to prolonged food deprivation that depletes body fat stores (Dickerman et al. 1993). It is important, however, to consider appetitive behaviors, precurrent behaviors that reflect internal motivation (Craig 1917). In addition to the consummatory aspects of sex behavior, hamsters display appetitive behaviors. During the infertile phase of the estrous cycle, they tend to be aggressive toward males, but as ovulation approaches they become more tolerant and begin to solicit males by placing vaginal scent marks near the entrance to their burrow. Sexual motivation in hamsters is determined by counting the number of aggressive bites and the number of vaginal scent marks made in response to adult male hamsters or male hamster odors (Johnston 1974, 1975; Johnston 1977). If female hamsters make decisions about foraging vs. mating in a 90-min period at dusk, we wondered whether such decisions are made the day before ovulation, when hamsters might be able to fine-tune their paracopulatory behaviors according to subtle changes in prior energetic status. After many years of studying the anestrus-inducing effects of severe energetic challenges, such as total food deprivation in lean hamsters, we examined the effects of mild energetic challenges with no obvious effect on the HPG system. For example, prior to these experiments, our traditional paradigm for inducing anestrus was borrowed from Professor Lawrence Morin and involved 48 h of food deprivation beginning on days 1 and 2 of the 4-day estrous cycle in very lean hamsters (Morin 1986). If hamsters are lean (85–95 g in body weight) and the period of deprivation encompasses the morning after ovulation and ends 2 days before ovulation, anestrus is virtually guaranteed. In contrast, anestrus is not induced in fatter female hamsters (greater than 104 g) or in females food deprived later in the estrous cycle, after the ovarian follicle has already reached maturity (Schneider et al. 2007). To the contrary, after 48 h of total food deprivation, fatter hamsters show normal 4-day estrous cycles and robust estrous behavior (i.e., the arched-back, immobile posture, lordosis, with the same duration and latency as *ad libitum*-fed females in response to an adult, sexually-experienced male). Although the consummatory behavior, lordosis, is not affected, their appetitive behaviors are decreased by food deprivation (Fig. 1; Schneider et al. 2007). Despite continued estrous cycles and robust, short latency, long-duration lordosis in fatter females, this level of food deprivation significantly decreases the number of vaginal scent marks and increases the latency to show aggressive behavior compared to the females fed *ad libitum* (Fig. 1A and B, Schneider et al. 2007). In addition to changing their appetitive sex behavior, food-deprived hamsters increase the appetitive ingestive behavior, food hoarding, ten to a hundred fold (Buckley and Schneider 2003; Schneider et al. 2007). Furthermore, the mean plasma estradiol concentrations in the food-deprived and *ad libitum*-fed groups are not significantly different (Fig. 1C). These results bring to light the relevance of (1) energetic challenges that do not significantly inhibit the HPG system and consummatory sex behavior and (2) appetitive sex and ingestive behaviors.

## Changes in the activation of RFRP-3 cells are closely associated with changes in appetitive behaviors during fasting and re-feeding

It is not surprising that females are less interested in courtship when they are hungry; however, the neuroendocrine mechanisms that set behavioral priorities under mild energetic challenges are unknown. Furthermore, these temporary effects on behavioral priorities might be a critical, yet overlooked function of the so-called “feeding” hormones and neuropeptides. The Kriegsfeld and Schneider laboratories hypothesized that priorities for sex and ingestive behaviors might be set by changes in the activation of cells that secrete RFamide-related-peptide-3 (RFRP-3), the mammalian ortholog of avian gonadotropin-inhibitory-hormone (GnIH).

Avian GnIH and Syrian hamster RFRP-3 are both dodecapeptide members of the RFamide peptide family, a family composed of molecules with an RFamide (Arg-Phe-NH2) motif at the C-terminus. GnIH was first isolated from the brains of Japanese quail and was found to have the amino acid sequence, SIKPSAYLPLRFamide. The molecule was named GnIH because it acted directly on the quail pituitary to inhibit the secretion of gonadotropins (Tsutsui et al. 2000). Subsequently, it was discovered that cDNAs of mammalian species encoded three homologous peptides, RFRP-1, 2, and 3.

Of these three RFRPs, RFRP-2 is not an RFamide peptide, and RFRP-1 does not inhibit gonadotropin secretion. RFRP-3, however, injected intracerebroventricularly (i.c.v.) reduces plasma LH in a variety of mammalian species (reviewed by Bentley et al. (2010)), including ovariectomized Syrian hamsters (Kriegsfeld et al. 2006). In sheep and birds, the effects occur directly on the pituitary (Tsutsui et al. 2000; Clarke and Parkington 2014), whereas in rodents, the effect occurs indirectly by inhibition of gonadotropin-releasing hormone (GnRH) (Kriegsfeld et al. 2006; Ducret et al. 2009; Wu et al. 2009). The amino acid sequence of RFRP-3 in Syrian hamsters is TLSRVPSLPQRFamide (Tsutsui 2010). Thus, the mammalian gene for RFRP-3 in relation to the quail gene for GnIH is considered to be an ortholog, a homologous gene that diverged after a speciation event, and in which part of the gene and its main function are conserved.

We hypothesized that RFRP-3 might set behavioral priorities such that ingestive behavior is favored over sex behavior. This idea is consistent with the following considerations. First, increases in GnRH-I and II increase sex behavior and/or inhibit ingestive behavior (Dorsa and Smith 1980; Dorsa et al. 1981; Mackay-Sim and Rose 1986; Temple et al. 2003; Kauffman et al. 2005; Barnett et al. 2006), and RFRP-3 in mammals and GnIH in birds both inhibit GnRH secretion. This has been shown with regard to inhibition of GnRH secretion by winter day lengths (reviewed by Clarke and Smith (2010); Henningsen et al. (2016); Kriegsfeld et al. (2015)), stressful stimuli (Iwasa et al. 2014; Son et al. 2014b; Geraghty et al. 2015; Kim et al. 2015; Clarke et al. 2016; Ernst et al. 2016), and energetic deficits (Clarke et al. 2012; Fraley et al. 2013c; Leon et al. 2014; Lynn et al. 2015). Finally, in a wide variety of species, RFRP-3 administration inhibits aspects of reproductive behavior (Bentley et al. 2006; Johnson et al. 2007; Piekarski et al. 2013) and stimulates food intake (Johnson et al. 2007; Clarke et al. 2012). Information about the effects of RFRP-3 on appetitive behavior was lacking, and we therefore examined association between appetitive behavior and this peptide.

RFamide peptide levels in the brain are difficult to measure, but the function of these peptides can be inferred by measuring the activation of cells that contain the peptide. Using immunohistochemistry, cells are double-labeled for RFRP-3 immunoreactivity (RFRP-3-Ir) and Fos-like immunoreactivity (Fos-Ir). Fos is the protein product of the immediate-early gene, *c-fos*, an established marker for cellular activation correlated with neurohormone/neurotransmitter secretion (Curran and Morgan 1987; Hoffman et al. 1993a, 1993b). The activation of RFRP-3-Ir cells is estimated by the percent of RFRP-3-Ir cells double-labeled with Fos-Ir. Thus, a collaborative line of experiments in the Kriegsfeld and Schneider laboratories examined the effects of different durations of mild food restriction on the activation of cells labeled for RFRP-3-Ir.

In the Schneider laboratory, Candice M. Klingerman used a unique preference apparatus to measure behavioral priorities in groups of Syrian hamsters that were food restricted for 0, 4, 8, or 12 days, and two more groups food-restricted for 12 days and then returned to *ad libitum* food intake for 4 or 8 days. In the Kriegsfeld laboratory, Wilbur P. Williams, III, measured the activation of RFRP-3-Ir cells in the DMH of the same groups of hamsters. Female subjects were housed in a burrow system. The home cage was attached to a tunnel that, when opened, led to a T-shaped intersection leading to two more tunnels that led in the opposite directions. The home cage was at the bottom of the T, the food was at the end of the tunnel on the left arm, and a sexually-experienced male hamster was at the end of the tunnel on the right-hand arm of the T. Using this apparatus, female hamsters were provided the option of spending time with food or spending time with the male, and we calculated their “male preference” defined as ([the time spent with males minus time spent with food] divided by the total time). The food-restricted group received 75% of their baseline daily intake of standard rodent chow. The timing of food restriction was scheduled so that tests for behavior occurred on the third day of the estrous cycle (peak vaginal scent marking) and the fourth day of the estrous cycle (the day of lordosis and ovulation). All tests occurred at the onset of the dark phase of the photoperiod, and behaviors were scored in real time by the experimenter every 5 s for 15 min. The female subjects continued to have access to the arms of the apparatus for the next 75 min. At the end of the 75 min (90 min total), the experimenter weighed the food in the home cage and food source box to determine the amount of food hoarded and eaten. On Day 4 of the estrous cycle, females were sacrificed and the experimenters took a terminal blood sample and perfused the females. The brains were fixed, frozen, and prepared for immunohistochemical (IHC) double-labeling for RFRP-3-Ir and Fos-Ir.

**Fig. 2:**
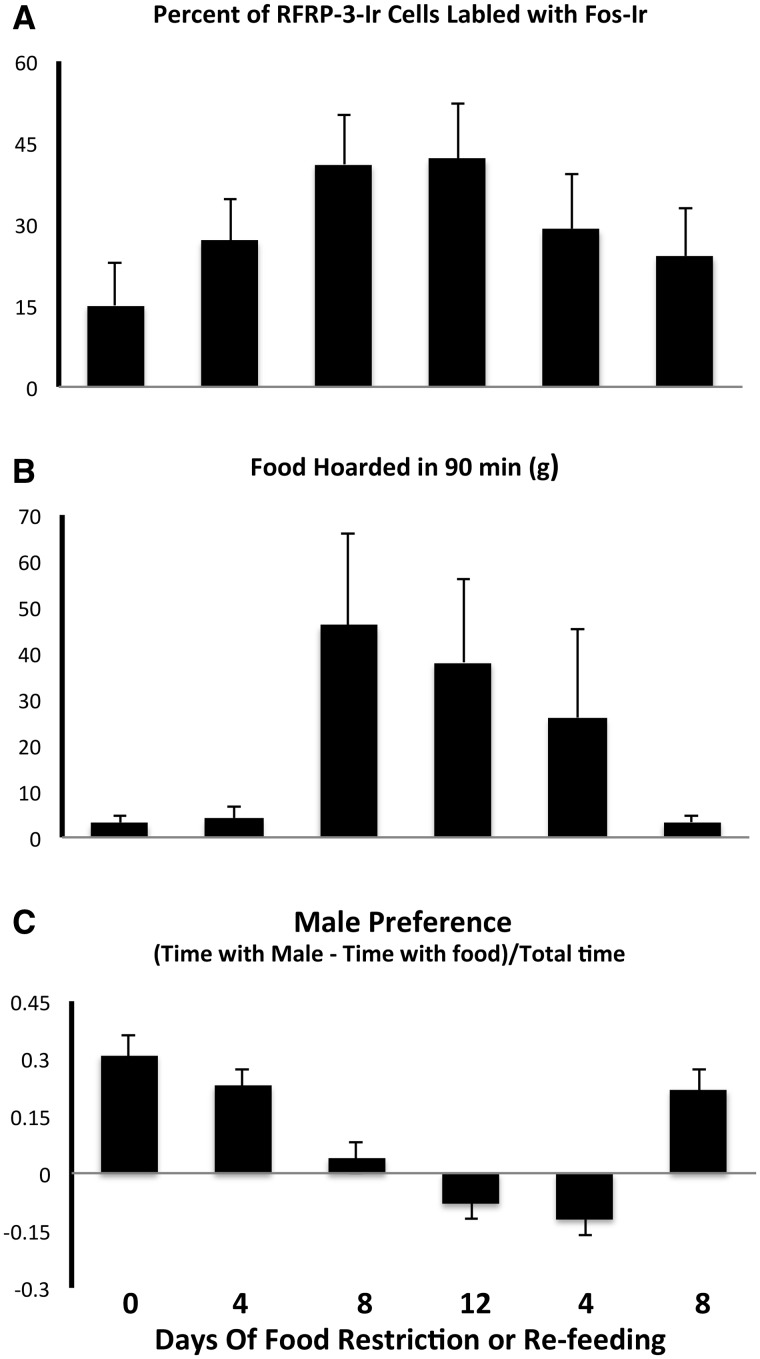
Mean and standard error of the mean for (**A**) the percent of RFamide-Related Peptide-3-immunoreactive (RFRP-3-Ir) cells labeled with Fos-like Immunoreactivity (Fos-Ir), (**B**) amount of food hoarded in 90 min, (**C**) male preference (amount of time spent with males minus the time spent with food) divided by the total time in groups of hamsters either food restricted or fed *ad libitum* for 0, 4, 8, and 12 days or food restricted for 12 days and re-fed for 4 or 8 days. *Significantly different from *ad libitum* at *P* < 0.05 (Adapted from Klingerman et al. 2011).

In Syrian hamsters, RFRP-3-Ir is restricted to the DMH. In the DMH of food-restricted females, there was a gradual increase in the activation of RFRP-3-Ir cells concomitant with an increase in the duration of food restriction and the level of food hoarding. The level of cellular activation and food hoarding peaked at 12 days after the start of food restriction, and gradually decreased at 4 and 8 days after the start of *ad libitum* food availability (Fig. 2A). These gradual changes in activation of RFRP-3-Ir cells were remarkably similar to the changes in appetitive ingestive behavior (food hoarding, Fig. 2B) and were the exact opposite of changes in appetitive sex behavior (Fig. 2C, Klingerman et al. 2011b). These changes in activation of RFRP-3-Ir cells and appetitive behavior occurred despite the fact that there was no significant effect of food restriction on plasma levels of estradiol, progesterone, food intake during the 90-min period, 24-h food intake, or lordosis frequency and duration (Klingerman et al. 2010; Klingerman et al. 2011b).

## Elevation of RFRP-3 in the brain is sufficient to induce food hoarding and inhibit sexual motivation

When RFRP-3 is infused i.c.v. in both *ad libitum*-fed and mildly food-restricted female Syrian hamsters, in *ad libitum*-fed, estrous cycling females, RFRP-3 infusion significantly decreases the preference for males vs. food and increases appetitive ingestive behavior (food hoarding), with no significant effect on consummatory aspects of ingestive behavior (food intake or body weight). Food restriction in vehicle-treated and RFRP-3-treated females decreases male preference and significantly increases food hoarding on the post-ovulatory day of the estrous cycle, and infusion of RFRP-3 does not exaggerate the effects of food restriction (Benton et al. 2017). The effect on both behaviors is statistically significant, but the effect sizes (eta-squared values) are greater for sexual motivation than for food hoarding. Furthermore, RFRP-3-induced food hoarding does not approach the level of food hoarding induced by food restriction, whereas the male preference in RFRP-3-treated females was similar to that seen in females food restricted for 8 days. These results are consistent with a role for RFRP-3 in food restriction-induced changes in appetitive behaviors, especially appetitive sex behaviors. These results are also partially consistent with RFRP-3 effects found in experiments that used different behavioral paradigms. For example, continuous i.c.v. infusion of RFRP-3 in *ad libitum*-fed female Syrian hamsters decreases the number of vaginal scent marks in response to cues from an intact male compared to the number of vaginal scent marks in response to cues from a castrated male (Piekarski et al. 2013). This result emphasizes a decrease in sexual motivation, specifically, rather than a decrease in general sociality or curiosity. This result is consistent with the effects of RFRP-3 on copulatory behaviors in some species (Bentley et al. 2006; Johnson et al. 2007; Ubuka et al. 2014), but not in others (Clarke et al. 2012). It is important to note that appetitive behaviors were not measured in these studies on other species. The stimulatory effect of RFRP-3 on food hoarding is partially consistent with effects of RFRP-3 on ingestive behavior in other species. With both acute and long-term treatment, central elevation of RFRP-3 levels increases food intake in mice, rats, sheep, non-human primates, drakes, and chicks (Tachibana et al. 2005; Johnson et al. 2007; Murakami et al. 2008; Tachibana et al. 2008; Clarke et al. 2012; Fraley et al. 2013). In other studies, measurements are typically limited to the consummatory aspects of ingestive behavior, and it is unknown therefore whether RFRP-3 would have increased the appetitive aspects of ingestive behavior, such as speed of eating or consumption of an unpalatable diet (common measures of ingestive motivation).

It is clear from these RFRP-3 infusions studies that elevation of intracerebral RFRP-3 is sufficient for changes in appetitive behaviors in female Syrian hamsters (Benton et al. 2017). It is not clear, however, whether RFRP-3 action is necessary for effects on appetitive behavior, and effects of RFRP-3 antagonists are under investigation.

## The behavioral effects of ovarian steroids are masked by *ad libitum* feeding

**Fig. 3:**
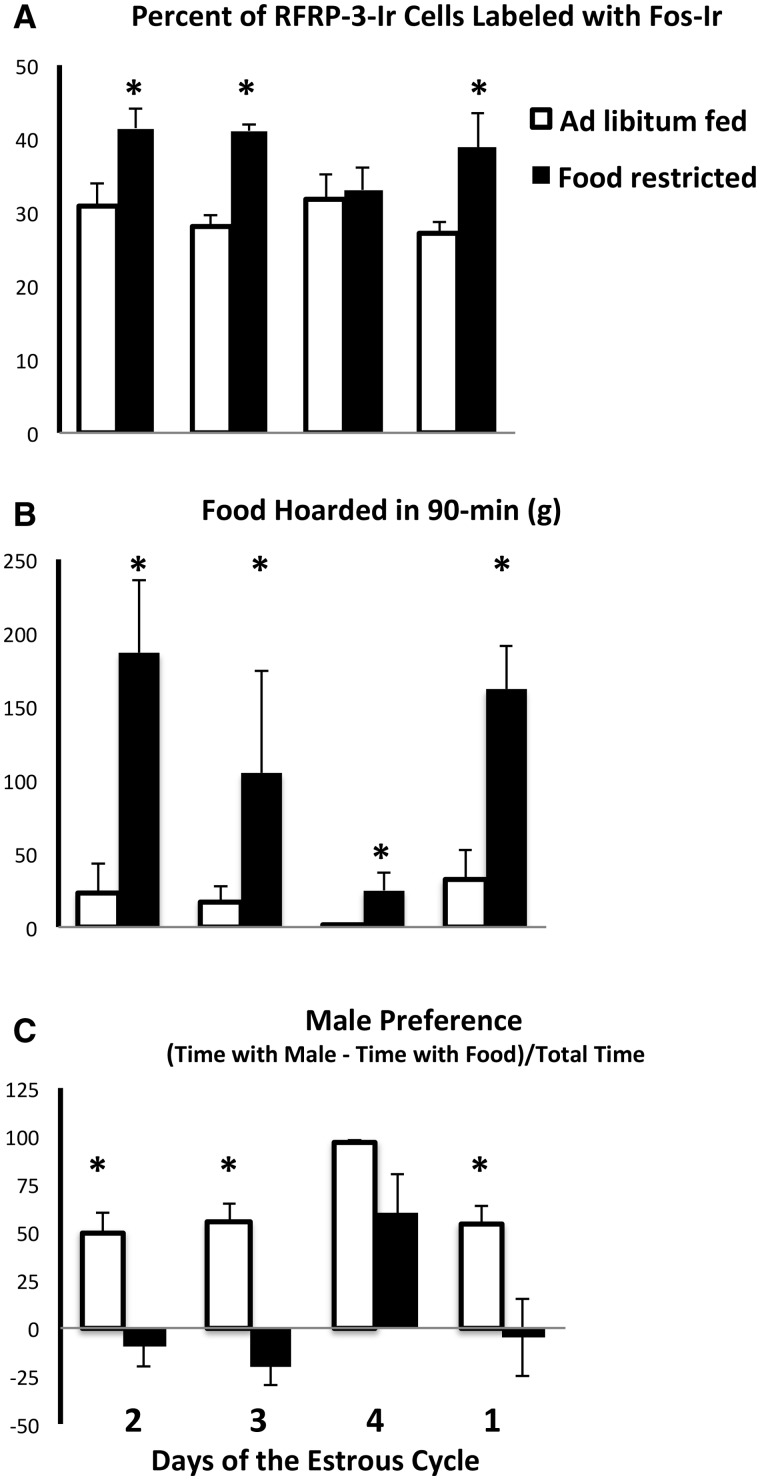
Mean and standard error of the mean for (**A**) the percent of RFamide-Related Peptide-3-immunoreactive (RFRP-3-Ir) cells labeled with Fos-like Immunoreactivity (Fos-Ir), (**B**) the amount of food hoarded in 90 min, (**C**) male preference (amount of time spent with males minus the time spent with food) divided by the total time across the 4-day estrous cycle in female Syrian hamsters either fed *ad libitum* or food-restricted to 75% of their *ad libitum* intake for 8 days. Tissue was collected from groups representing every day of the 4-day estrous cycle. *Significantly different from *ad libitum* at *P* < 0.05 (Adapted from Benton et al. 2017 and Klingerman et al. 2010).

When the females’ preference for males vs. food is observed every day of the 4-day estrous cycle, the 4-day pattern of behavior differs according to the availability of food and mates. Females fed *ad libitum* opt to spend more time with males than food on all 4 days of the estrous cycle (Fig. 3C, Klingerman et al. 2010). In contrast, food-restricted females shift their behavioral priorities. They spend most of their time hoarding food until Days 3 and 4 of the estrous cycle, when they shift back to a preference for visiting the male (Fig. 3C). This follows the pattern of ovarian steroid secretion over the estrous cycle, in which estradiol rises on the evening of Days 3 and 4 of the estrous cycle, falls precipitously after ovulation, and remains low until the evening of Day 3 (Shaikh 1972). Thus, in mildly food-restricted females housed in the presence of a male, there emerges a clear fluctuation in motivation that resembles the well-known fluctuations in ovarian steroid levels. The opposite pattern occurs with ingestive behavior. A mild level of food restriction, i.e., 75% of *ad libitum* intake, stimulates food hoarding (Fig. 3B) on the infertile days of the cycle, but these food-restriction-induced effects are absent at the time of estrus (Schneider et al. 2007; Klingerman et al. 2010; Klingerman et al. 2011a, 2011b; Abdulhay et al. 2014). In summary, when food availability is unlimited, the effects of fluctuating ovarian hormones on behavior are masked, and when the female is subjected to mild food restriction, the effects of ovarian steroids on behavior are revealed in sharp relief (Fig. 3B and C). Subsequent experiments were designed to test the hypothesis that this pattern was associated with patterns of activation of RFRP-3-Ir cells in the female hamster DMH.

## Effects of estrous cycles on behavioral priorities are matched by changes in the activation of RFRP-3-Ir cells

Given the striking similarity in appetitive ingestive behavior and activation of RFRP-3-Ir cells (Fig. 2), we hypothesized the following: In mildly food-restricted females, when circulating concentrations of ovarian steroids are low, RFRP-3-Ir cells are activated and RFRP-3 secretion promotes food hoarding. Furthermore, we hypothesized that RFRP-3-Ir cell activation is modulated by high levels of ovarian steroids during the hours before ovulation. This inhibits the urge to forage and hoard food and stimulates sexual motivation, thereby synchronizing mating with the time of highest fertility. To test this idea, activation of RFRP-3-Ir cells in the DMH was measured over the 4 days of the estrous cycle in food-restricted and *ad libitum*-fed females.

As predicted, food restriction-induced increases in activation of RFRP-3-Ir cells occur on only the three non-estrous days of the estrous cycle, and do not occur on the day of ovulation (Fig. 3A, Benton et al. 2017). Given that kisspeptin is an RFamide peptide that is stimulatory for GnRH and sex behavior and is responsive to energetic status ((Paul et al. 2009; Ladyman and Woodside 2014) and reviewed by (De Bond and Smith 2014)), it might be expected that kisspeptin-immunoreactive cells would be inhibited by food restriction. Contrary to expectation, no such pattern occurs in the activation of kisspeptin-immunoreactive cells (Benton et al. 2017). There is no main effect of either food restriction or the day of the estrous cycle (or interaction) on the activation of kisspeptin-immunoreactive cells. These data implicate changes in cellular activation of RFRP-3-Ir, but not kisspeptin-Ir, in the food-restriction-induced changes in motivation in female Syrian hamsters.

## Ovarian steroids modulate food restriction-induced activation of RFRP-3-Ir cells

Changes in behavior over the estrous cycle suggest that ovarian steroids are involved. It has long been known that ovarian hormones influence both ingestive and sex behaviors. Estradiol treatment, for example, inhibits food intake and facilitates sexual motivation (reviewed by Asarian and Geary (2006), Clegg (2012), Santollo and Daniels (2015), Santollo et al. (2013), Wade and Gray (1979), Wade and Schneider (1992)). In Syrian hamsters, ovariectomy exaggerates food restriction-induced increases in the preference for food vs. sex, and in mildly food-restricted, ovariectomized females, the switch to preference for males can be induced by exogenous treatment with estradiol and progesterone (Klingerman et al. 2010). Furthermore, the effects of ovarian steroids are masked by unlimited food availability. In *ad libitum*-fed, ovarian steroid-treated, ovariectomized females, the preference for males is high, but not that much higher than that of ovariectomized, vehicle-treated females. In ovariectomized females, mild food restriction drastically decreases preference for males and increases food hoarding (but not food intake) in vehicle-treated but not in ovarian steroid-treated females (Klingerman et al. 2010). We therefore hypothesized that the increases in ovarian steroids at the time of estrus modulate restriction-induced increases in the active of RFRP-3-Ir cells in the DMH. To test the idea, females were treated with one of three steroid regimens chosen because of their known effects on appetitive and consummatory sex and ingestive behavior: (1) estrus-inducing levels of estradiol followed 48 h later by progesterone (6 h before testing), (2) estradiol alone (6 h before testing), or (3) progesterone alone (6 h before testing). Increases in RFRP-3-Ir cell activation are seen in ovariectomized females treated with vehicle, but not in females treated with estradiol followed by progesterone or in females treated with progesterone alone. In contrast, treatment with estradiol alone does not decrease RFRP-3 cell activation. The modulation of RFRP-3-Ir activation by estradiol plus progesterone and progesterone alone support our hypothesis, but the lack of effect of estradiol alone does not fit with the well-known estradiol modulation of RFRP-3 cells via estrogen receptor (Kriegsfeld et al. 2006). This lack of estradiol blockade of food-restriction-induced activation of RFRP-3-Ir is interpreted with caution because, at other doses and time courses, estradiol alone might blunt the effects of food restriction on the activation of RFRP-3-Ir. The effects of estradiol plus progesterone and progesterone alone are consistent with the idea of ovarian steroid modulation of food-restriction-induced increases in the activation of RFRP-3-Ir cells (Benton et al. 2017).

Progesterone, which can come from the brain and/or ovary, facilitates lordosis in Syrian hamsters and contributes to the positive feedback that underlies the gonadotropin surge in rats (Buntin and Lisk 1984; Micevych et al. 2008). Progesterone alone (in addition to progesterone plus estradiol) prevents the food restriction-induced increase in RFRP-3 cell activation, and we therefore examined whether RFRP-3 cells contain progestin receptor-immunoreactivity (PR-Ir). Whereas plentiful RFRP-3-Ir is present in the DMH and PR-Ir is observed in the ventromedial hypothalamus (VMH) and basal medial hypothalamus, including the arcuate nucleus of the hypothalamus (Arc), no RFRP-3-Ir is present in the VMH or Arc, no PR-Ir is present in the DMH, and no PR-Ir is colocalized with RFRP-3-Ir (Benton et al. 2017). Thus, if progesterone modulates RFRP-3 cell activation, it might act independently of progestin receptors (perhaps via rapid membrane action) or via intermediate projections from progesterone receptor containing cells located in other brain areas (such as the Arc or the VMH). For example, neuropeptide Y-immunoreactivity (NPY-Ir) projections are found in close apposition to RFRP-3-Ir cells (Klingerman et al. 2011b), and NPY cells are known to contain progestin receptors (Dufourny et al. 2005).

## Are behavioral priorities set by cues from food availability specifically or by energy availability in general?

The total energy availability is presumably the sum of energy derived from ingested food plus energy that can be mobilized from tissue triglycerides and glycogen, minus total energy expenditure. To determine whether the observed changes in behavior are caused by the availability of food specifically or energy in general, we manipulated the females’ need to expend energy by either housing female hamsters at cold ambient temperatures or by allowing them to engage in voluntary exercise. In the first experiment, groups of estrous-cycling hamsters were fed *ad libitum* while they were housed at either 5 °C (cold) or 22 °C (control), and then tested for appetitive and consummatory behavior for 90 min on each day of the estrous cycle. In females housed at 22 °C, high levels of sexual motivation and low levels of food hoarding were seen every day of the estrous cycle (Abdulhay et al. 2014), similar to the pattern that emerged from the experiments of Klingerman et al. (2010). In contrast, in females housed at 5 °C, high levels of food hoarding and low levels of sexual motivation were seen on three out of the four estrous cycle days, similar to the pattern that emerged in food-restricted females. High levels of sexual motivation were restricted to the peri-ovulatory day in both cold-housed females fed *ad libitum* and warm housed, food-restricted females. On the three non-estrous days, these females showed high levels of food hoarding, but not food intake. In a second experiment, a separate cohort of females housed at 22 °C was divided into two groups: One group was housed with and the other without access to running wheels for 8 days. Later, when these two groups were tested in the preference apparatus, females housed with running wheels showed low levels of appetitive sex behaviors on three out of the 4 days of the estrous cycle, and high levels of sexual motivation restricted to the peri-ovulatory day, similar to the pattern of sexual motivation seen in cold-housed females and in food-restricted females. These changes in behavior occurred despite no significant inhibitory effects of cold ambient temperature or access to running wheels on the serum levels of estradiol or progesterone (Abdulhay et al. 2014). We noted one interesting difference between females with running wheels and females cold-housed. Unlike cold-housed females, those with running wheels showed low levels of food hoarding and high levels of food intake. Access to running wheels appeared to decrease the motivation to hoard food while increasing the motivation to eat food, an unexpected outcome that is worthy of further investigation.

In general, all three energetic challenges (food restriction, housing at cold temperatures, and increased wheel running) unmask the estrous cycle fluctuations in sexual motivation that are obscured in laboratory conditions, i.e., isolation in a small cage with an overabundance of food (Abdulhay et al. 2014). This is highly significant because it suggests that behavioral neuroendocrinologists miss clues to the underlying mechanisms when they study animals isolated from opposite-sex conspecifics with unlimited food availability and few behavioral options.

## How do decreases in food availability communicate with RFRP-3 cells and behavior?

It can be speculated that RFRP-3 cells that influence motivation might receive information about metabolic fuel availability by detectors of glucose, fatty acid oxidation, or both. These signals are thought to travel from peripheral tissues to the hypothalamus via the caudal brain stem via vagal and or autonomic afferents. The effects of specific pharmacological inhibitors of metabolic fuel availability on RFRP-3 cells and appetitive behavior have not been examined to the best of our knowledge, but would be informative. Another possibility is that information about fuel availability is communicated by a circulating factor. RFRP-3 cells are leptin responsive, although only a small percentage colocalize with leptin receptor (Poling et al. 2014; Rizwan et al. 2014). Furthermore, levels of the adipocyte hormone, leptin, fall rapidly with food restriction and are lower in lean compared to fat Syrian hamsters (Schneider et al. 2000), and treatment with leptin inhibits food restriction-induced increases in food hoarding in Syrian (Buckley and Schneider 2003) and Siberian hamsters (Keen-Rhinehart and Bartness 2008). Concentrations of leptin fall with food restriction and are negatively correlated with the percent of RFRP-3 cellular activation in Syrian hamsters subjected to 25% food restriction (Benton et al. 2017). Leptin concentrations do not, however, fluctuate across the estrous cycle in food-restricted or *ad libitum*-fed females, and thus, the ovarian steroid modulation of RFRP-3 are most likely not mediated by effects of ovarian steroids on serum leptin concentrations (Benton et al. 2017). One possibility worthy of future investigation is the idea that circulating levels of leptin interact with the level of metabolic fuel availability (e.g., Schneider and Zhou 1999).

## How do RFRP-3 cells alter behavioral motivation?

A number of other chemical messengers might be involved in effects of energy availability on sexual motivation and hunger for food, and some of these might interact with the RFRP-3 system. Many chemical messengers change with fasting and re-feeding and treatment with these chemical messengers influences consummatory behavior and the HPG system (reviewed by Schneider et al. (2013)). They are well-studied with regard to the consummatory ingestive behavior, but few of these have been studied with respect to appetitive ingestive and sex behaviors. Examples include kisspeptin, NPY, agouti-regulated protein (AgRP), glucocorticoids, and corticotropin-releasing hormone (CRH).

With regard to kisspeptin, we find no association between appetitive behaviors and activation of kisspeptin-Ir over the estrous cycle (Benton et al. 2017). This alone should not be taken as strong evidence against a role for this peptide in control of behavior, and, other evidence supports a role for kisspeptin in metabolic control of the HPG system and behavior (Castellano et al. 2005; Fernandez-Fernandez et al. 2006; Kalamatianos et al. 2008; Stengel et al. 2011; Clarke 2014; De Bond and Smith 2014; Ladyman and Woodside 2014). For example, kisspeptin is implicated in the interaction between food availability and photoperiod in seasonally breeding Siberian hamsters (Paul et al. 2009). Furthermore, food restriction might change the response to kisspeptin rather than secretion of kisspeptin (Wahab et al. 2008).

In addition, another hypothalamic peptide, NPY, has been implicated in the preference for sex vs. food (Ammar et al. 2000). Furthermore, chronic infusion of RFRP-3 increases activation of NPY cells in sheep and increases expression of NPY in rats (Clarke et al. 2012). Elevated levels of intracerebral NPY are both necessary and sufficient for food deprivation-induced increases in food hoarding behavior in Siberian hamsters (Day et al. 2005; Dailey and Bartness 2009). In Syrian hamsters, NPY terminals project to the DMH and show close apposition to RFRP-3 cells (Klingerman et al. 2011b). Together, these results suggest that RFRP-3 might work downstream from NPY cells that are influenced by low energy availability.

There are many other plausible mediators of the effects of energy availability on motivation, including melanocortin receptor agonists and antagonists, alpha-melanocyte-stimulating hormone, agouti-related peptide (AgRP), and melanin concentrating hormone (MCH). MCH, NPY/AgRP, and vasoactive-intestinal peptide send projections to GnRH cells in mice (Ward et al. 2009), and at least some types of GnRH-producing cells might have direct stimulatory effects on sexual behavior as well as inhibitory effects on food intake (Dorsa and Smith 1980; Dorsa et al. 1981; Mackay-Sim and Rose 1986; Temple et al. 2003; Kauffman et al. 2005; Barnett et al. 2006). These chemical messengers might act downstream from RFRP-3 or act independently of RFRP-3 to influence appetitive sex and ingestive behaviors.

In addition to NPY and the melanocortins, the stress hormones are obvious candidates for mediation of RFRP-3 action on behavior. Food restriction, even mild food restriction, might be categorized as a stressor and might be expected to increase the secretion of hormones of the hypothalamic-pituitary-adrenal system (Stewart et al. 1988). Furthermore, in rats, RFRP-3 cells are colocalized with glucocorticoid receptor (GR) (Kirby et al. 2009) and the promoter region of the RFRP-3 gene contains two glucocorticoid response elements (GREs) (Son et al. 2014). Immobilization stress, which increases circulating glucocorticoid concentrations, increases the total number of RFRP-3 cells in birds and rats (Calisi et al. 2008; Kaewwongse et al. 2011). Furthermore, this treatment induces RFRP-3 expression in rats, and this effect is removed by adrenalectomy (Kirby et al. 2009), consistent with the idea that glucocorticoids are necessary for stress-induced expression of RFRP-3. Thus, it is plausible that energetic challenges act as stressors that increase glucocorticoids, which activate RFRP-3-Ir cells.

In Syrian hamsters, however, effects of energetic challenges on estrous behavior (lordosis) are more directly related to increases in intracerebral CRH than with glucocorticoids (Jones et al. 2002; Seymour et al. 2005). In Syrian hamsters, antagonists to CRH are potent facilitators of lordosis in response to estradiol and progesterone (Jones et al. 2002), and this might reflect a role for CRH in sexual motivation in Syrian hamsters that would influence the choice between food hoarding and courtship. The role of glucocorticoids and CRH in the effects of mild energetic challenges on appetitive behaviors is worthy of investigation.

## Summary

Throughout the animal kingdom in species from many vertebrate and invertebrate taxa, individuals make trade-offs when resources are constrained. When energy availability is a limiting factor, animals trade-off their investment in reproductive output to engage in ingestive behaviors that are necessary for homeostasis in oxidizable metabolic fuels. The amplitude of oscillations in ingestive and sex behavior can be large, and the period of these oscillations can be long, e.g., 365 days in some aquatic mammals, or short, e.g., 4 days in Syrian hamsters. Furthermore, the pattern of behavioral fluctuation and its link to hormones can be masked by unlimited food supplies, small enclosures, and a lack of opposite-sex conspecifics. This is important because it suggests that behavioral neuroendocrinologists are blinded to the underlying mechanisms when they study animals isolated from opposite-sex conspecifics, with unlimited food availability, and with few behavioral options. A growing body of data collected in food-restricted females housed in a semi-natural burrow system supports the idea that RFRP-3-steroid interactions orchestrate the changes in behavioral priorities that occur over the estrous cycle. A hypothetical model has been proposed to explain this phenomenon in Syrian hamsters subjected to mild energetic challenges, i.e., mild compared to total food deprivation used to study metabolic control of the HPG system. This model was formulated with regard to a hypothetical female in the wild that must expend energy to ingest energy and that has options regarding interactions with opposite-sex conspecifics.

The model can be described as follows. For most of the estrous cycle, in a female subjected to mild energetic challenges, when circulating concentrations of ovarian steroids are low, RFRP-3 cells are activated and RFRP-3 secretion and action is increased to promote appetitive ingestive behaviors, such as foraging and food hoarding. RFRP-3 cell activation is modulated by high levels of ovarian steroids during the hours before ovulation, and this inhibits the urge to forage and hoard food and stimulates sexual motivation, thereby synchronizing mating with the time of highest fertility. The model is supported by the fact that, during most of the estrous cycle, mild food restriction increases food hoarding and decreases sexual motivation, and this effect is reversed by re-feeding (Klingerman et al. 2010; Klingerman et al. 2011a, 2011b). I.c.v. treatment with RFRP-3 mimics these effects in *ad libitum*-fed females. Food restriction-induced increases in food hoarding and decreases in sexual motivation occur only on the non-estrous days of the estrous cycle or in ovariectomized females treated with vehicle (Klingerman et al. 2010). Similarly, food restriction-induced increases in activation of RFRP-3-Ir cells occurs on only the three non-estrous days of the estrous cycle or in ovariectomized females treated with vehicle (Benton et al. 2017). Food restriction-induced RFRP-3-Ir cell activation does not occur on the day of estrus and ovulation. Furthermore, the food restriction-induced activation of RFRP-3-Ir in ovariectomized females is blocked by pre-treatment with progesterone alone or estradiol plus progesterone (Benton et al. 2017). These results add to a growing body of data (e.g., Piekarski et al. 2013) implicating RFRP-3 in the neural circuitry that underlies natural changes in behavioral motivation over the estrous cycle in animals with limited energy availability and access to potential mating partners. The attention to appetitive behavior is critical, because, had we measured only consummatory behaviors (lordosis and food intake), the link to RFRP-3 would have been missed. This set of experiments emphasizes the importance of appetitive behaviors and their function in the habitats in which the species live and evolve. Together, these experiments implicate ovarian steroids and RFRP-3 in the trade-off between ingestive and sex behavior when energy availability is constrained.
